# Effects of schedule exercise therapy on chronic insomnia

**DOI:** 10.1097/MD.0000000000030792

**Published:** 2022-09-23

**Authors:** Yuan-Gao Liao, Feng-Zhen Huang, Xiao-Hong Ni, Hong-Yan Ke, Yu Tian, Mei Yu, Guo Jin, Gui-Hai Chen

**Affiliations:** a Sleep Medical Center and Department of Neurology, Huanggang Central Hospital, Huanggang, China; b Institute of Transitional Medicine at University of South China, Chenzhou, China; c Department of Neurology, the First People’s Hospital of Chenzhou, Chenzhou, China; d Department of Neurology (Sleep Disorders), the Affliated Chaohu Hospital of Anhui Medical University, Hefei (Chaohu), China.

**Keywords:** chronic insomnia, medication, schedule exercise therapy

## Abstract

Schedule exercise therapy (SET) is a novel nonpharmacological intervention for the treatment of chronic insomnia disorder (CID). The aim of this study was to explore the effects of SET on CID. Methods: One hundred and eighteen CID were recruited and randomized into medication (MED) or medication combined with SET (MSET) groups. Over 12 observational weeks, sleep and mood status were evaluated using the Pittsburgh Sleep Quality Index (PSQI), Insomnia Severity Index (ISI), Epworth Sleepiness Scale (ESS), Self-rating Depression Scale (SDS), and Self-rating Anxiety Scale (SAS). At the end of the observational period, the rates of clinically effective hypnotic use were calculated. At 12 weeks, the PSQI progressively decreased for all subjects combined (*P *< .001) as well as ISI (*P *< .001), ESS (*P *< .001), SDS (*P *< .001), and SAS (*P *< .001). The decreases in PSQI (*P *< .05), ISI (*P *< .05), SDS (*P *< .01), and SAS (*P *< .05) in the MSET group were significantly larger than those in the MED group, but not the same as those in the ESS group (*P *> .05). At the trial endpoint, the clinically effective rate was significantly higher (*P *< .05) and the hypnotic usage rate was lower (*P *< .05) in the MSET group than in the MED group. SET may be an effective treatment for insomnia in patients with CID.

## 1. Introduction

Chronic insomnia disorder (CID) is characterized by persistent difficulty in initiating or maintaining sleep for at least 3 nights a week for 3 months, resulting in impaired daytime function and significant distress.^[1]^ It has been estimated that 10% to 30% of the general population worldwide suffer from symptoms of insomnia,^[2-5]^ and approximately 6% of the general population meets the diagnostic criteria of insomnia.^[6,7]^ A growing number of sleeping pills are available to treat or manage insomnia, such as benzodiazepines or non-benzodiazepine γ-aminobutyric acid receptor agonists, sedative antidepressants, melatonin agonists, antihistamines, orexin receptor antagonists, anti-epileptic drugs, atypical antipsychotics, and over-the-counter agents.^[8]^ However, patients with CID are typically reluctant to receive medication (MED) therapy for insomnia due to side effects and dependency risks.

Non-pharmacological approaches to insomnia management are acceptable or precedent for patients with CID, and many of these approaches have proven to have an equivalent effect on sleep MEDs.^[9-13]^ Cognitive behavioral therapy is the primary non-pharmacological approach recommended as the preferred treatment for insomnia (CBT-I) based on European and American guidelines.^[14,15]^ Since Spielman first proposed the concept of behavioral treatment for insomnia,^[16]^ an increasing number of studies have examined its mechanism of action and clinical applications.^[17-21]^ Clinical research has demonstrated that CBT-I can be a safe and effective treatment option for patients with CID with various medical and psychiatric comorbidities, including chronic pain, fibromyalgia, breast cancer, and vasomotor symptoms in perimenopausal women.^[13,22-26]^

However, CBT-I is underutilized in patients with CIDs. The reasons for this include high initial cost, limited consulting time, insufficiently trained technicians, and ineffective referral pathways.^[27-29]^ To fill the gap in limited consultation, exploration studies of digitally delivered CBT-Is have been conducted. These studies found that digitally delivered CBT-I may be a viable alternative^[5,21,30,31]^ although there are barriers between CID patients and medical providers across developing countries.

For these reasons, we propose Schedule Exercise Therapy (SET) as a novel non-pharmacological treatment for CID patients. The principles of the CBT-I include psychological education, relaxation training, sleep restriction, stimulus control, and cognitive therapy. SET entailed the core principles of CBT-I, such as stimulus control, sleep restriction, and chronotherapy.^[16]^ Additionally, SET integrates body exercise into its treatment paradigm. In contrast to CBT-I, SET summarizes all of its key elements into a simple and concise table, which enables SET recipients to adhere to the treatment plan. Here, we introduce the SET protocol and report our clinical exploration of its therapeutic effect on CID at our hospital. It is important to note that in this insomnia treatment study, we prescribed antidepressants for patients with CID since insomnia and depression were inseparable and bidirectional in causation.

## 2. Materials and Methods

### 2.1. Participants

We recruited outpatients^[32]^ from the Sleep Medical Center of our hospital between August 2020 and July 2021. All patients were diagnosed with CID according to the criteria of the International Classification of Sleep Disorders, 3rded.^[33]^ Participants were excluded if they: were night shift workers; had comorbid severe medical diseases; had other sleep disorders such as sleep-wake rhythm disorders, sleep breath disorder, and restless legs syndrome; had taken antidepressants or antipsychotics in the past 3 months or were pregnant, lactating, or planning to become pregnant. The study was approved by the Ethics Committee of the Huanggang Central Hospital.

### 2.2. Design

**Figure 1.**
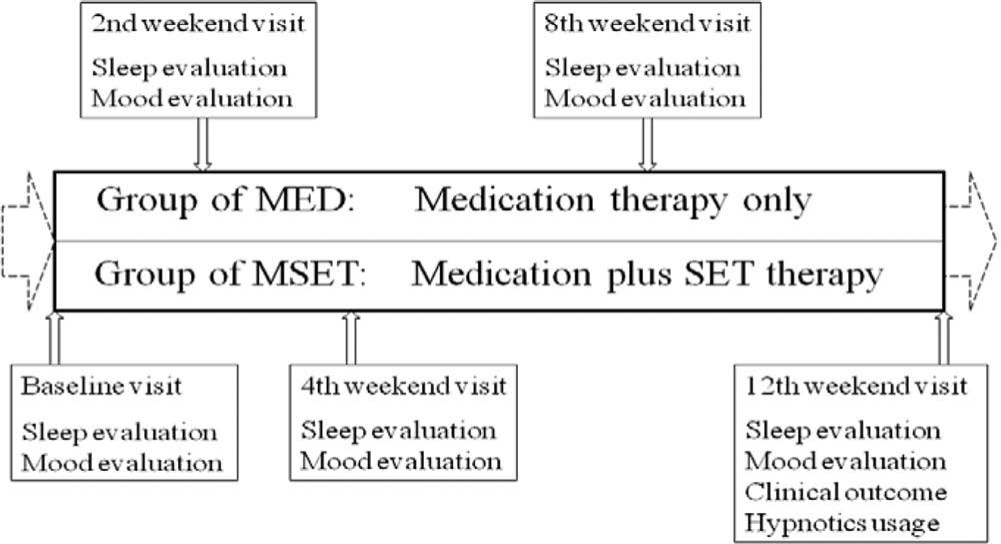
Flowchart of the 12-week treatment and follow-up.

The CID patients were recruited and randomized into 2 groups: MED treatment and medication combined with SET treatment (MSET). The patients received a treatment paradigm (Fig. 1) with systematic treatment for 12 weeks in the MED or MSET conditions. The sleep and mood status of the patients were assessed at baseline and on the 2nd, 4th, 8th, and 12th weekends. The rates of clinically effective hypnotic use were also analyzed at the 12th follow-up appointment.

### 2.3. Medication treatment

**Figure 2.**
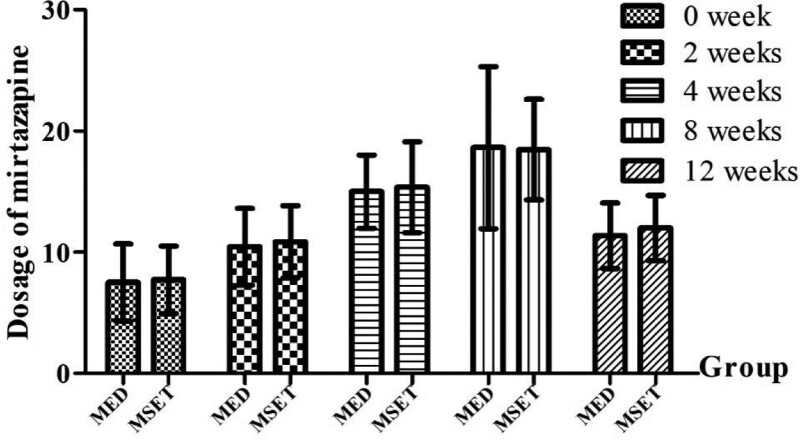
The use of mirtazapine. The dosage of mirtazapine had no significant difference (*P* > .05) between the MED group and MSET group in 0 week, 2 weeks, 4weeks, 8 weeks, and 12 weeks. MED = medication, MSET = medication combined with SET.

MED therapy, in its basic framework, consists of antidepressants and hypnotics. Representative antidepressants were sertraline titrating from an initial dose of 50 mg/d to a maximum dose of 150 mg/d with an average dose of 100 mg/d, venlafaxine titrating from an initial dose of 75 to 150 mg/d, escitalopram oxalate 10 mg/d, and mirtazapine titrating from an initial dose of 7.5 mg/d to a maximum dose of 30 mg/day with an average dose of 15 mg/day. It is worth noting that 11 participants in the MED group and 10 in the MSET group were treated with mirtazapine. The dose of mirtazapine was not significantly different between the 2 groups (*P* > .05), as shown in Figure 2. The reasons for choosing the above antidepressant drugs were as follows: they are widely used, convenient to use, and have relatively few side effects. The representative hypnotics were as follows: “eszopiclone” 1 to 3 mg/d, “zopiclone” 3.75 to 7.5 mg/d, “zolpidem” 5 to 10 mg/d, and “estazolam” 1 to 2 mg/d. All hypnotics were prescribed before nighttime sleep for <5 days per week.

### 2.4. Schedule exercise therapy

Participants in the MSET group were given SETs in addition to MED(s). Table 1 shows the SET protocol (HG-202008-03), which was developed by the Sleep Medical Center of Huanggang Central Hospital.

**Table 1:** Protocol of schedule exercise therapy (HG-202008-03).

Time	Items	Notes
6:30	Getting up	Quit sleep environment* immediately
6:30—8:00	Morning exercise	Running or brisk walking recommended
8:00—12:00	Working	Positive and absorbed working demanded
13:00—14:00	Midday activity	Indoors recreational activities recommended
14:00—17:30	Working	Positive and absorbed working demanded
20:00—21:00	Evening exercise	Balls game or dancing recommended
22:30	Going to bed	Enter sleep environment* immediately

* Sleep environment: quiet, light avoiding and delightful cool.

The principle of SET is to schedule a regular time for most patients’ daily life activities and physical exercise. The concrete steps were as follows: getting up at 6:30, performing morning exercise between 6:30 and 8:00, working from 8:00 to 12:00, doing midday activity from 13:00 to 14:00, working from 14:00 to 17:30, doing evening exercise from 20:00 to 21:00, and going to bed at 22:30. The SET protocol makes specific requirements relevant to every activity such as a quiet sleep environment. In summary, SET can be phrased as “one foundation and two points;” the one foundation is a helpful sleep environment, and the 2 points are strict schedule and intensified body exercise. As to the exercise, we recommended moderate-intensity physical exercise. SET is an overall management of everyday life through a set of compulsory regulations and guidelines, so it can be suitable for treating almost all kinds of insomnia, except for night shift workers and physically weak people. We helped every participant adjust the mobile phone alarm at a fixed time for the timing instructions.

### 2.5. Sleep evaluation

The Pittsburgh Sleep Quality Index (PSQI), Insomnia Severity Index (ISI), and Epworth Sleepiness Scale (ESS)^[15]^ were used to evaluate sleep quality. The PSQI is a 19-item questionnaire used to measure sleep quality and disturbance over the previous month. The total score is 21; a score of <7 indicates good sleep quality, whereas a score of ≥7 indicates poor sleep quality.^[34]^ The ISI is a 7-item self-report instrument used to assess insomnia symptoms and consequences. The total score is 28, and a score ≤7 indicates no insomnia. A higher score indicates greater insomnia severity.^[35]^ The ESS is an 8-item self-administered questionnaire that evaluates the tendency to doze off during the day. The total score is 24, and a score >10 indicates significant daytime sleepiness.^[36]^

### 2.6. Mood evaluation

The self-rating depression scale (SDS) and self-rating anxiety scale (SAS) were used to evaluate mood symptoms. The SDS and SAS are 2 norm-referenced scales that reflect psychological and physiological symptoms. The SDS was used to assess depressive symptoms, whereas SAS was used to assess anxiety symptoms. Both scales included 20 items, ranging from 0 to 4 points each, which were then converted to a full score of 100. A score >50 was considered abnormal, indicating more apparent depression and anxiety.^[37,38]^

### 2.7. Clinical outcome measurement

Based on the changes in sleep status and the reductive ratio of PSQI score compared to the baseline, we defined the clinical outcomes of insomnia as: clinical remission indicated by the disappearance of insomnia symptoms and a PSQI reductive ratio of more than 75%; marked effectiveness indicating that the sleep status significantly improved and the PSQI score reductive ratio was more than 50%; effective indicating that the sleep status improved and the PSQI score reductive ratio was more than 25%; and ineffective, indicating that the sleep status had no improvement or worsened and the PSQI score reductive ratio was <25%.

### 2.8. Statistical analysis

Categorical data were expressed as proportions, and continuous data were expressed as mean ± standard deviation. We used the chi-square test of equal proportions to compare the categorical variables between the 2 groups. We used Student’s *t* test to compare the variables between the groups and 2-way repeated measures ANOVA to compare the variables between the baseline and follow-up visits. The analysis utilized intent-to-treat population and last‐observation‐carried‐forward analysis methods for missing data. The data were tabulated electronically using Microsoft Office Excel and analyzed using the IBM Statistical Package for the Social Sciences (SPSS version 21.0). A *P* value of < .05 or < .01 as considered to be statistically significant.

## 3. Results

### 3.1. Characteristics of participants groups

**Figure 3.**
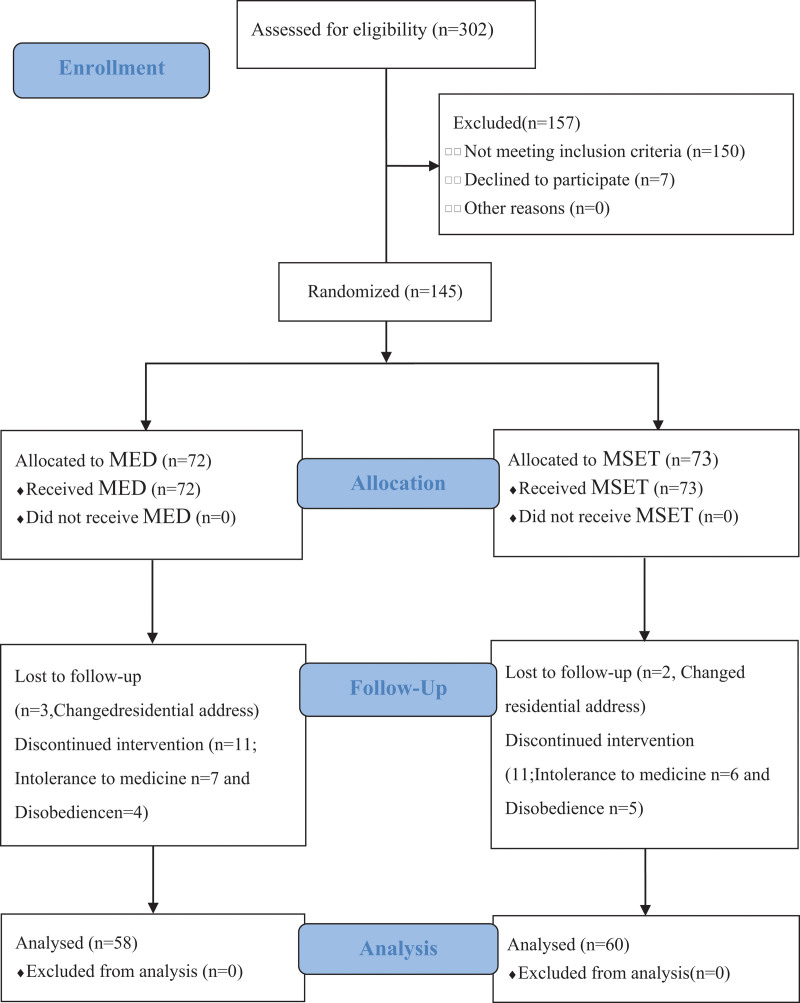
Flow diagram.

A consort chart describing the participants from enrollment to the completion of the study is shown in Figure 3. A total of 302 patients with CID were registered and 145 were eligible for randomization. Of the 145 randomized patients (72 in the MED group and 73 in the MSET group), 7 patients from the MED group and 6 from the MSET group withdrew due to MED intolerance. Most patients adhered to the treatment protocol, and only 4 from the MED and 5 from the MSET group were excluded. Finally, 3 and 2 patients from the MED and MSET groups, respectively, were lost to follow-up.

The participants’ characteristics are shown in Table 2. Of the 150 patients who did not meet the inclusion criteria, 31 were night shift workers, 49 had comorbid severe medical diseases, 23 had other sleep disorders, 29 had taken antidepressants or antipsychotics in the past 3 months, and 18 were pregnant or planning to become pregnant. Among the remaining enrolled patients, 23 in the MED group and 25 in the MSET group had comorbidities, such as hypertension and diabetes, and there was no significant difference between the 2 groups. There were no significant differences in sleep disturbance or depression between the 2 groups.

**Table 2:** Characteristics of patients in both the groups.

Terms	MED	MSET	*P* value
Numbers	58	60	
Age	47.7 ± 13.9	44.3 ± 13.9	.197
Sex			
Male (%)	22 (37.9)	18 (30.0)	.438
Female (%)	36 (62.1)	42 (70.0)	
Occupation			
Physical (%)	25 (43.1)	26 (43.3)	1.000
nonphysical (%)	33(56.9)	34(56.7)	
Duration (months)	13.3 ± 7.5	11.5 ± 7.0	.181
Sleep disturbances			
PSQI	16.83 ± 2.72	17.37 ± 2.31	.248
ISI	24.31 ± 2.88	24.42 ± 2.64	.835
ESS	7.91 ± 3.96	8.17 ± 2.89	.692
Mood			
SDS	62.85 ± 7.25	62.03 ± 5.95	.458
SAS	63.31 ± 7.55	62.34 ± 8.25	.465

ESS = Epworth sleep scale, ISI = insomnia severity index, MED = medication, MSET = medication combined with SET, PSQI = Pittsburgh sleep quality index, SAS = self-rating anxiety scale, SDS = self-rating depression scale.

### 3.3. Sleep

**Figure 4.**
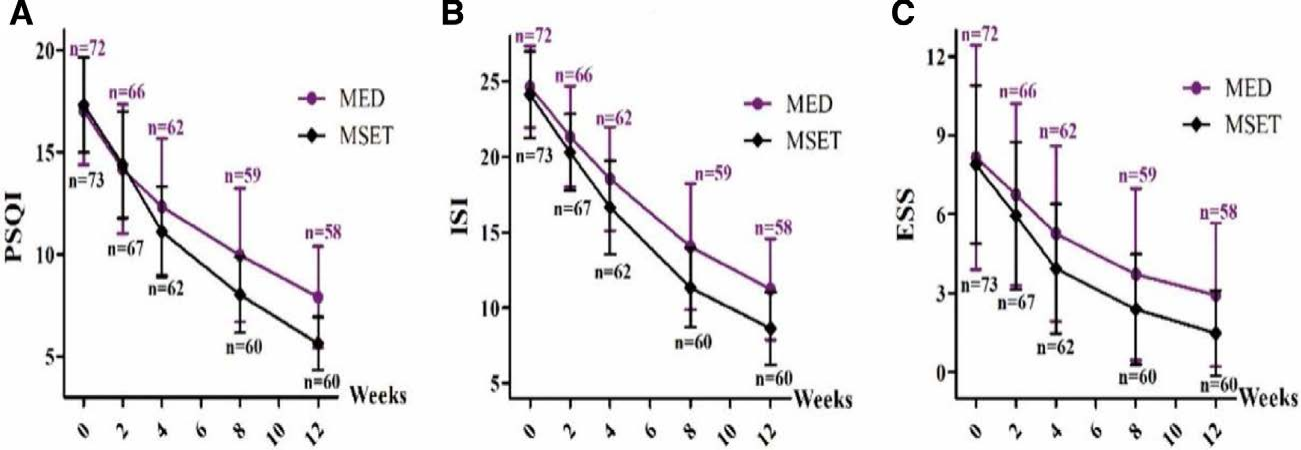
Dynamic changes of the scores of sleep-related scales during the 12-week observation period. The scores of Pittsburgh Sleep Quality Index (PSQI, A), Insomnia Severity Index (ISI, B), and Epworth Sleep Scale (ESS, C) progressively decreased in both MED and MSET groups (*P *< .001). The decreased degree of PSQI (A) and ISI (B) were significantly larger in the MSET group (*P *< .05), but not the same as the scores of ESS (C, *P *> .05). MED = medication, MSET = medication combined with SET.

The PSQI, ISI, and ESS scores obtained at baseline and at the 2nd, 4th, 8th, and 12th^[32]^ follow-up weekends are shown in Table 3. The PSQI, ISI, and ESS scores in both the MED and MSET groups decreased gradually during the 12-week study, as shown in Figure 4.

**Table 3:** Results of sleep and mood evaluation through whole observation.

Scales	Groups	Visit time points				
		Baseline	2nd week	4th week	8th week	12th week
PSQI (score)	MED	16.83 ± 2.71	14.19 ± 3.18	12.48 ± 3.41	10.02 ± 3.26	7.91 ± 2.49
	MSET	17.37 ± 2.31	14.35 ± 2.62	11.12 ± 2.24	8.03 ± 1.86	5.63 ± 1.29
ISI (score)	MED	24.31 ± 2.88	21.14 ± 3.49	18.53 ± 3.54	14.09 ± 4.22	11.24 ± 3.35
	MSET	24.41 ± 2.64	20.55 ± 2.20	16.67 ± 3.16	11.35 ± 2.62	8.63 ± 2.41
ESS (score)	MED	7.91 ± 3.96	6.76 ± 3.51	5.36 ± 3.34	3.72 ± 3.28	2.93 ± 2.73
	MSET	8.17 ± 2.89	6.12 ± 2.80	4.00 ± 2.47	2.38 ± 2.10	1.48 ± 1.62
SDS (score)	MED	62.67 ± 7.25	59.37 ± 6.57	57.75 ± 7.78	53.23 ± 8.45	50.87 ± 7.09
	MSET	61.86 ± 5.95	58.55 ± 5.39	54.01 ± 6.16	50.16 ± 6.13	45.96 ± 6.33
SAS (score)	MED	63.13 ± 7.55	59.50 ± 8.09	57.50 ± 8.55	53.32 ± 8.99	51.36 ± 7.42
	MSET	62.17 ± 8.29	58.20 ± 7.80	54.47 ± 7.85	50.01 ± 6.46	46.15 ± 6.33

ESS = Epworth sleep scale, ISI = insomnia severity index, MED = medication, MSET = medication combined with SET, PSQI = Pittsburgh sleep quality index, SAS = self-rating anxiety scale, SDS = self-rating depression scale.

Repeated-measures ANOVA showed that the PSQI scores progressively decreased with visits for all subjects [*F*_(4, 580) _= 613.570, *P *< .001], but the degree of decline in the MSET group was significantly larger than that in the MED group [*F*_(1580) _= 6.808, *P *< .05]. The ISI scores also gradually decreased with visits for all patients [*F*_(4, 580) _= 808.562, *P *< .001]; however, the MSET group showed a more prominent decline than the MED group [*F*_(1, 580) _= 12.478, *P *< .05]. The ESS score gradually decreased with visit points for all patients combined [*F*_(4, 580) _= 229.999, *P *< .001], but the degree of decrease between the MED and MSET groups was not significantly different [*F*_(1, 580) _= 3.877, *P *> .05], as shown in Figure 4.

### 3.4. Depression and anxiety

**Figure 5.**
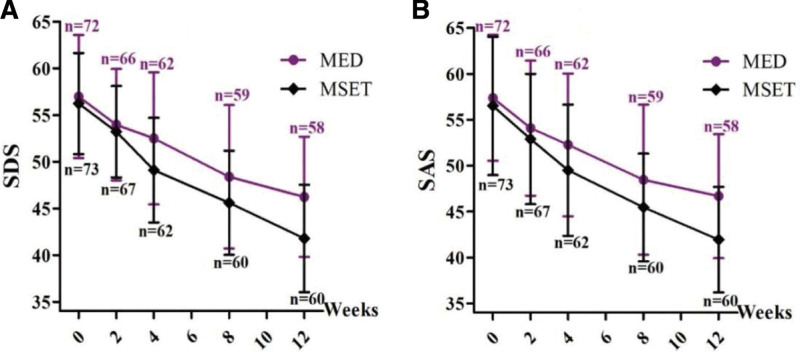
Dynamic changes of the scores of affective scales during the 12-week observation period. The scores of Self-rating Depression Scale (SDS, A) and Self-rating Anxiety Scale (SAS, B) gradually decreased in both MED and MSET groups (*P *< .001), but the degree of SDS (A) and SAS (B) decline were significantly larger in the MSET group (*P *< .05). MED = medication, MSET = medication combined with SET.

The SDS and SAS scores obtained at the baseline the 2nd, 4th, 8th, and 12th follow-up weekends are shown in Table 3. At baseline, SDS and SAS scores in both groups decreased gradually throughout the observational period. Repeated-measures ANOVA showed that both SDS [*F*_(4, 580) _= 165.266, *P *< .001] and SAS [*F*_(4, 580) _= 103.730, *P *< .001] scores progressively decreased with visits for all subjects combined, and the decreased degree of SDS [*F*_(1, 580) _= 7.164, *P *< .01] and SAS [*F*_(1, 580) _= 5.521, *P *< .05] in the MSET group was significantly greater than that in the MED group (Fig. 5).

### 3.5. Clinical outcome

The clinical outcomes measured at the endpoint are shown in Table 4. The MSET group had a significantly higher clinical remission rate (*x*^2 ^= 5.642, *P *< .05) and marked-effective rate (*x*^2 ^= 18.829, *P *< .05) than the MED group.

**Table 4:** Clinical outcomes at study endpoint.

Group	Case	Remission	Marked- effective	Effective	Ineffective
Medication	58	1	39	15	3
Medication plus SET	60	8*	51**	1	0

SET = schedule exercise therapy.

* *P *< .05;

** *P *< .01.

### 3.6. Hypnotic usage

At the endpoint visit of 12-week observation, we defined the participants who took hypnotics more than 2 days in the last week as “hypnotics usage group” and showed as Table 5. The hypnotic usage rate in the MSET group was significantly lower than that in the MED group (*x*^2 ^= 6.039, *P *< .05).

**Table 5:** Hypnotics usage at the last visit point.

Group	Case	Usage	No usage	Percent (%)
Medication	58	20	36	34.48
Medication plus SET	60	9	51	15*

SET = schedule exercise therapy.

* *P *< .05.

## 4. Discussion

CBT-I is the preferred treatment for insomnia based on both the European and American guidelines.^[14,15]^ However, CBT-I is intrinsically inconvenient and hinders most patients with insomnia from receiving treatment, especially in developing countries.^[18,27-29]^ Thus, we introduced a non-pharmacotherapy named SET, which was partially derived from CBT-I, to treat insomnia disorders. To test the effects of our proposed SET on CID, we conducted a 12-weeks research.

Our study showed that the PSQI, ISI, ESS, SDS, and SAS scores in both the MED and MSET groups were all significantly lower across the observational period, which indicated that MED or MED plus SET therapy could effectively ameliorate sleep and affective symptoms. At the end point of MED vs MSET groups, the scores of PSQI were decreased from 16.83 ± 2.71 to 7.91 ± 2.49 versus from 17.37 ± 2.31 to 5.63 ± 1.29, the scores of ISI were decreased from 24.31 ± 2.88 to 11.24 ± 3.35 versus from 24.41 ± 2.64 to 8.63 ± 2.41, the scores of SDS were decreased from 62.67 ± 7.25 to 50.87 ± 7.09 versus from 61.86 ± 5.95 to 45.96 ± 6.33, and the scores of SAS were decreased from 63.13 ± 7.55 to 51.36 ± 7.42 versus from 62.17 ± 8.29 to 46.15 ± 6.33. The above results showed that the MSET group had a significantly greater decrease in PSQI, ISI, SDS, and SAS scores than the MED group. The results showed that compared with CBT-I, SET could also improve sleep quality in a simpler and easier way, which compensates for the shortcomings of CBT-I, such as high initial cost and limited consulting time. In addition, the insomnia remission and marked-effective rates were significantly higher, and the hypnotics usage rate was significantly lower in the MSET group than in the MED group when evaluated at the endpoint. Taken together, we conclude that SET plus therapy could enhance MED effects in patients with CID by not only improving sleep quality but also relieving symptoms of depression and anxiety. However, the ESS scores was used to assess sleepiness during the day, and the scores were decreased from 7.91 ± 3.96 to 2.93 ± 2.73 versus from 8.17 ± 2.89 to 1.48 ± 1.62 at the end point of MED vs MSET groups, which revealed the negative slope of ESS scores was similar in both groups. The possible reasons were as follows: PSQI and ISI refer to the quality of sleep and severity of insomnia, and ESS refers to sleepiness during the daytime by self-assessment. Our results indicate that SET could improve night sleep better than daytime sleepiness. Moreover, the high subjectivity and high expectations of the participants also affected the ESS result.

We speculated that there are several ways to explain the effect of SET on chronic insomnia. SET could improve sleep efficiency by limiting 8 hour of time in bed and accumulating sleep debt during the daytime. Samuel et al found in a Drosophila model that flies could not maintain effective sleep when given an overabundance of sleep because of sleep fragmentation and a mismatch in circadian timing. Otherwise, sleep opportunity and ability could better match sleep restriction in both wild-type and mutant flies. In addition, sleep restriction improved the daytime sleep. These results are similar to those in humans.^[39]^ SET could help to establish the bed-sleep stimulus control by creating a sleep environment and limiting it for sleep only. SET might help remodel the sleep-wake rhythm by scheduling everyday living activities and maintaining activity throughout the day. Jade et al discovered that sleep time relative to the circadian phase and irregular sleep patterns caused poor functional outcomes in delayed sleep-wake phase disorder, which demonstrated that the sleep-wake rhythm is important for insomnia.^[40]^ Kandeger et al found that circadian rhythm differences could indirectly affect food addiction through elevated insomnia and impulsivity, indicating that circadian rhythm is closely related to insomnia and impulsivity.^[41]^ In addition to these 3 active components of the non-pharmacological treatment of CID, there are still other merits of SET. SET encourages patients to live positive and optimistic lives. SET can enhance the physical status of patients by incorporating exercise. This is in line with CBT-I in principle,^[16]^ but SET converted the key points of CBT-I into a simple set of instructions to facilitate and encourage effective clinical practice.^[15,42]^

The relationship between sleep and psychiatric disorders is inseparable and may lead to bidirectional causation.^[43,44]^ Insomnia is significantly associated with an increased risk of psychiatric disorders.^[45,46]^ Psychiatric comorbidities may worsen the sleep quality. Therefore, clinicians should consider psychiatric comorbidities when treating insomnia.^[47]^ In most insomnia patients, pathophysiological changes were changed in a similar way of depression, such as hyperfunctional hypothalamus-pituitary-adrenal axis, whole body and brain metabolic rate,^[48,49]^ increased secretion of inflammatory cytokines,^[50,51]^ and decreased brain-derived nerve growth factor.^[52,53]^ These factors are predictors of insomnia severity, daytime functional impairment, and prolonged chronic insomnia. Thus, in addition to non-pharmacological treatment, antidepressants were also a valuable treatment choice, and low dosage usually started to work. Antidepressants could restore these pathophysiological abnormalities, affect the symptoms of insomnia, and maintain long-term therapeutic effects.^[54,55]^ Moreover, our participants had mild-to-moderate depression and anxiety based on their scale scores. For these reasons, we prescribed a low dosage of antidepressants, such as sertraline, venlafaxine, escitalopram oxalate, and mirtazapin, for all participants in addition to hypnotics during the study period.

However, there are several limitations to our study, and further research is needed. First, it is suitable for nonphysical workers. We did not subgroup the insomnia patients into physical or nonphysical groups; therefore, we could not differentiate the exact effect of SET on physical workers. A meta-analysis revealed that regular exercise could exert moderate beneficial effects on sleep quality, but did not mention exercise intensity.^[56]^ Research has revealed that internationally recommended minimum levels of physical activity can improve daytime and nighttime symptoms of chronic insomnia, independent of daily light exposure.^[57]^ Thus, individualized treatment of SETs for physical workers is needed. Second, we did not adjust the clock time during the study period according to daylight saving time, as long daylight might interfere with the participants’ sleep-wake rhythm. A study showed that sleep-wake cycle quality has a stronger deterioration after spring compared with autumn transition, which demonstrates that the human circadian system adjusts to a phase delay more easily than to a phase advance.^[58]^ Thus, the clock time should be adjusted during the study period according to daylight saving time. Third, the lack of blinding of the investigators is also a limitation. Finally, because our clock time was only suitable for China, it should be adjusted according to different time zones around the world.

## Conclusions

SET should be a clinically available option for patients with CID to relieve insomnia and improve their affective conditions. Further studies are needed to examine the effectiveness of SET alone or as an add-on treatment for CID co-occurring with other medical conditions.

This work was financially supported by the National Foundation of Nature Science of China (81370444, 81671316), Huanggang Science and Technology Bureau (XQYF2021000005), and Science and Technology Development Projects of Chenzhou (No.zdyf2020048).

We thank LetPub (www.letpub.com) for linguistic assistance during the preparation of this manuscript.

## Author contributions

**Conceptualization:** Yuan-Gao Liao, Gui-Hai Chen.

**Data curation:** Feng-Zhen Huang, Xiao-Hong Ni, Yu Tian.

**Formal analysis:** Feng-Zhen Huang, Xiao-Hong Ni, Yu Tian.

**Funding acquisition:** Feng-Zhen Huang, Xiao-Hong Ni, Gui-Hai Chen.

**Investigation:** Yu Tian.

**Methodology:** Yu Tian.

**Project administration:** Mei Yua.

**Resources:** Mei Yua, Guo Jin.

**Software:** Hong-Yan Ke, Mei Yua, Guo Jin.

**Supervision:** Gui-Hai Chen.

**Validation:** Hong-Yan Ke, Guo Jin.

**Visualization:** Hong-Yan Ke, Guo Jin.

**Writing – original draft:** Yuan-Gao Liao.

**Writing – review & editing:** Yuan-Gao Liao, Feng-Zhen Huang.
